# Offset Printing Plate Quality Sensor on a Low-Cost Processor

**DOI:** 10.3390/s131114277

**Published:** 2013-10-24

**Authors:** Jelena Poljak, Guillermo Botella, Carlos García, Sanja Mahović Poljaček, Manuel Prieto-Matías, Francisco Tirado

**Affiliations:** 1 Faculty of Graphic Arts, University of Zagreb Getaldiceva 2, 10000 Zagreb, Croatia; E-Mails: jelena.poljak@grf.hr (J.P.); smahovic@grf.hr (S.M.P.); 2 Department of Computer Architecture and Automation, Complutense University of Madrid, 28040 Madrid, Spain; E-Mails: garsanca@ucm.es (C.G.); mpmatias@ucm.es (M.P.-M.); ptirado@ucm.es (F.T.)

**Keywords:** offset printing, embedded systems, low cost sensor, computer vision, ARM, Raspberry Pi

## Abstract

The aim of this work is to develop a microprocessor-based sensor that measures the quality of the offset printing plate through the introduction of different image analysis applications. The main features of the presented system are the low cost, the low amount of power consumption, its modularity and easy integration with other industrial modules for printing plates, and its robustness against noise environments. For the sake of clarity, a viability analysis of previous software is presented through different strategies, based on dynamic histogram and Hough transform. This paper provides performance and scalability data compared with existing costly commercial devices. Furthermore, a general overview of quality control possibilities for printing plates is presented and could be useful to a system where such controls are regularly conducted.

## 1. Introduction

**Figure 1.**
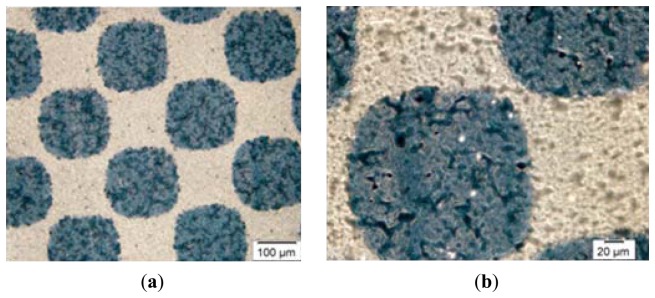
The surface structure of an offset printing plate.

Offset printing is currently the most commonly used printing technique. It is an indirect reproduction process, in which the printing ink is first transferred from the printing plate onto an intermediate carrier (e.g., blanket, rubber) and then onto the substrate. The principle of the offset printing process is based on the physicochemical differences between the printing (image) and the non-printing areas on the printing plate, which are practically on the same level (Figure 1). Between the printing and non-printing areas, there is a difference in height of 2–3 micrometers, a negligible difference compared with other printing techniques [1,2]. Printing areas are made of non-polar organic materials (creating a photosensitive layer) and have hydrophobic properties [3]. Separate from hydrophobic properties, printing areas have oleophilic characteristics, which mean that oil-based printing ink will adhere to them during the reproduction process. Non-printing areas are hydrophilic, repellant to oil-based inks, and made of mechanically- and electrochemically-grained aluminium oxide [4–7].

**Figure 2.**
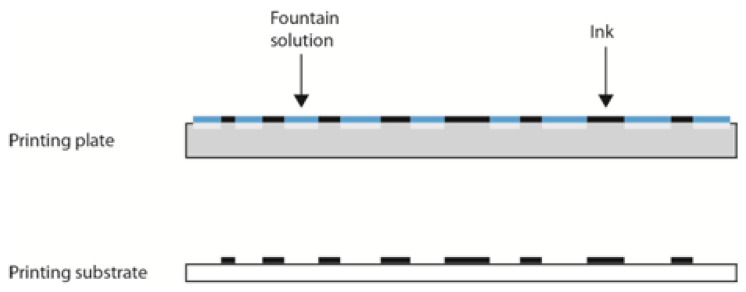
Offset printing plate after applying fountain solution and ink.

The printing process begins with the application of the fountain solution on the entire printing plate surface [8–10]. The fountain solution adheres only to the hydrophilic non-printing areas, which then become completely oleophobic. After the application of the fountain solution, the printing ink must be applied to the whole printing plate surface, where it adheres onto the printing areas and is transferred onto the printing substrate (Figure 2).

In order to obtain a certain quality of transferred ink on the substrate, every step in the reproduction process must be carefully controlled. Obviously, quality control is of major importance to the printing industry, where a procedure or set of procedures ensures that the printed product meets the quality criteria and requirements of the client. One method for ensuring quality is by measuring the printing plates, which provides a final opportunity for a visible inspection of the reproduced information before the printing process starts, since surface structures on the plates (printing and non-printing elements) will be responsible for adhering to the printing ink, which directly affects the visual appearance and the quality of the final product.

Quality control of a printing plate can be conducted by monitoring both surfaces—the printing and nonprinting areas—of the printing plate [10]. However, many factors must be taken into consideration while monitoring, such as the type of photosensitive layer, the surface structure of aluminium-oxide, the sensitivity and thickness of the photosensitive layer, the imaging device (the plate setter), the laser, the developing process, *etc.*, all of which can affect the quality level of those surfaces [9]. Detecting and correcting the deficiencies before the printing process starts eliminates several possible unexpected costs in reproduction.

**Figure 3.**
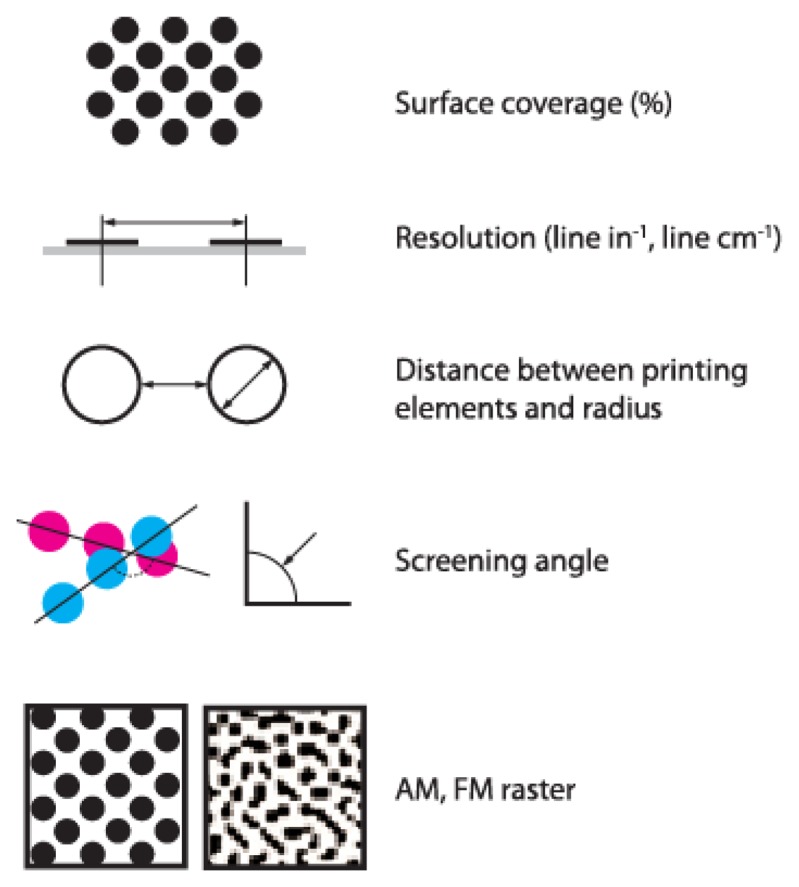
Quality defining parameters on offset printing plates.

Image quality on the printing plate surface is usually controlled by using different devices for measuring the quality level of the plates. These devices work on the principle of determining several parameters for monitoring its quality and are illustrated in Figure 3. One of the most controlled parameters is the surface coverage of printing elements [11–13]. This can be measured using different manual devices, which ensure quick and relatively constant results. At present, through the development of different application systems for image analysis, it is possible to define the surface coverage of printing elements based on the microscopic image of the surface [14].

The aim of this paper is to develop a new microprocessor-based sensor in order to determine the quality of the offset printing plate with circular shaped image elements through an introduction of different image analysis applications. This paper is based on a previous prototype [15] but contains several improvements to obtain real-time performance against commercial approaches. The hypothesis is that it is possible to define the surface coverage value of the printing element by using a new image analysis application, which can be implemented on a low-cost processor and used in practice. The implementation was made in order to evaluate the usage of portable devices used in practice with different image analysis solutions and to determine their usefulness for quality control in graphic arts technology. In addition to portable devices, the popular Matlab software used for image processing and image analysis was used in this evaluation.

## 2. Tools and Systems for Test Quality Measuring

### 2.1. Materials and Procedure

For this research, printing plate samples were made in the same processing conditions by copying a control wedge with a group of fields of 1%, 2%, 3%, 5%, 10%, 15% … to 100% coverage value. Printing plate samples were exposed with a metal halide lamp for 60 impulses. After the exposure, samples were developed in a commercial offset plate developer for a period of 15 s. The plate manufacturer defined these processing conditions for the workflow used [16, 17].

In order to define the printing plate's quality level, the surface coverage of the printing elements was observed. The surface coverage can be measured with devices designed for controlling the printing plates, which automatically calculate the coverage value, or by image analysis software. In this research, both methods (explained in Sections 3.1.1 and 3.1.2) for determining the surface coverage of printing elements were used.

Measurements of coverage values on the printing plate samples were performed by Gretag-Macbeth's IC Plate II plate reader [18–22] and repeated five times for each sample. The coverage values were shown on the display of the portable device and also in the supported software. In addition to this device, measurements were conducted using VipFLEX [23–25] and repeated five times for each sample. Moreover, to obtain images for defining the surface coverage values with image analysis software, the samples were captured and analysed using Matlab.

### 2.2. IC Plate II

**Figure 4.**
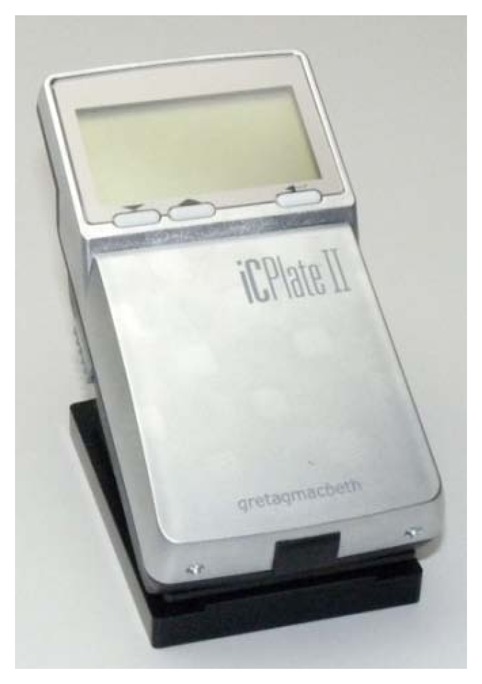
Gretag Macbeth's IC Plate II (image taken from http://www.idd.tu-darmstadt.de/ media/fachgebiet_idd/forschungdienstleistung/ausstattung_3/icPlate.jpg). Courtesy of Xrite Company (http://www.xrite.com/).

The Gretag-Machbeth IC Plate II (X-Rite—Corporate Headquarters, Grand Rapids, MI, USA) [18] is a portable plate reader that provides quick and accurate quality control (Figure 4). This device has automatic calibration, and the battery life is extended by LED illumination. The power-saving electronics and LED technology allow up to 30,000 measurements per battery set. The measuring time is 3–4 s. The measuring unit is calibrated automatically and is equipped with a high-resolution camera able to capture images from the printing plate surface.

The IC Plate II can measure a wide variety of plate types-metal and polyester, positive and negative, paper and foil. It can also measure and calculate surface coverage, the diameter of the printing element, screen resolution, and the screen angle. The built-in algorithm device eliminates dust and scratches as it reads the printing plate. Also, the screening algorithm allows measuring of a regular or stochastic type of raster. The IC Plate II uses the specially-developed plate measurement algorithm for determining surface coverage. The type of sensor used is CMOS 648 × 488 with a sensor resolution of 12,700 ppi.

### 2.3. VipFLEX

**Figure 5.**
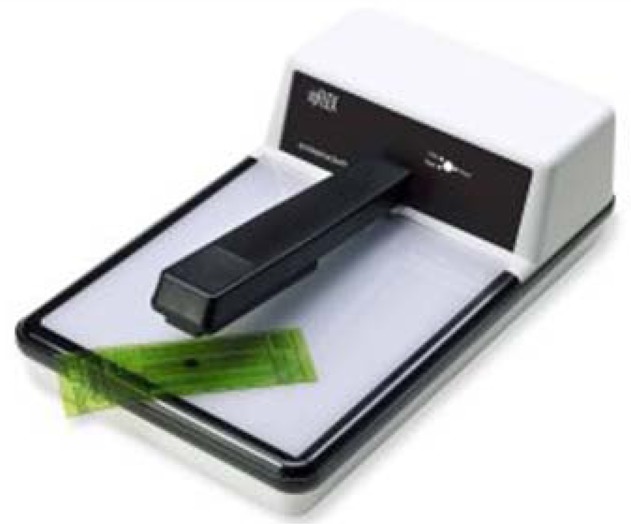
VipFLEX (image taken from http://www.mgvcolor.com/vipflex/). Courtesy of Xrite Company (http://www.xrite.com/).

VipFLEX is mainly intended for flexography plates, but it can also be used to measure paper, foils, film, and aluminium plates (Figure 5). This measuring system includes the VipFLEX desktop plate reader and PlateQualityFlexo software, characterized by a high-resolution camera with sophisticated image recognition. One battery set allows up to 50,000 measurements. VipFLEX can measure the dot area, dot size, edge factor, and mottling. It has the capability of measuring AM, FM, and hybrid screening. The device is equipped with an RGB sensor camera 640 × 480 with a resolution sensor of 10,000 ppi. Measuring time is very fast, less than 1 s. VipFLEX has two different measurement algorithms, chain dot and normal dot. The normal dot algorithm will produce the surface coverage value and contour factor, while the chain dot algorithm will only produce the surface coverage.

## 3. Methodology

### 3.1. Architecture of the System Proposed and Stimuli Used

In this section, the methods and strategies performed for measuring the quality of printing plates are described. As preliminary work, a study of measuring quality in printing plates is addressed by means of the high-level programming language software Matlab. This study is conducted in order to validate our algorithmic approach before formalizing the implementation of a low-cost sensor (Raspberry Pi board [26] and Carma-Kit from SECO Company (Arezzo, Italy) [27]) for quality estimation and degradation of printing plates.

Two strategies have been considered, the first one based on measuring the coverage ratio in the printing plates in an analogous way –*binarized quantification*–, as in commercial systems such as IC Plate [18] or VipFLEX [23]; and the second based on recognizing a basic shape (in this study, circles with a constant radius) and then suggesting a quality metric based on the deformation of the circles and the extrapolating forms affected by ambient noise –*printing element extraction and quantification*. In particular, our second strategy incorporates quality parameters used in printing quality, such as the resolution, the surface coverage, the distance between printing elements, and the screening angle.

#### 3.1.1. First Strategy (Binarized Quantification)

The quality of the printing plates can be determined in different ways, and one of those ways is by using computer vision algorithms. In determining the computer vision algorithms, the captured images of the printing plate surface were used. The computer vision algorithm, which was used in the first strategy, is less complex; in only three steps the results of the surface coverage were obtained, counting black and white pixels. This first strategy corresponds to our first approach, which aims to provide a quality metric based on the measurement of surface coverage in a percentage.

**Figure 6.**
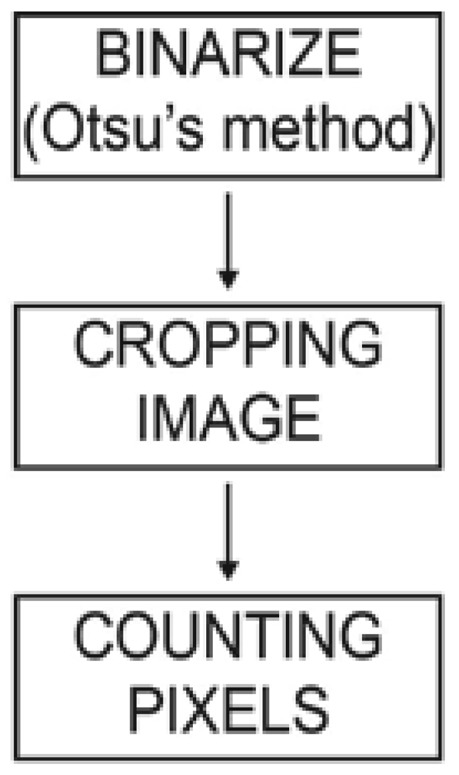
Flow chart from the first method implemented.

The *binarized quantification method* (Figure 6) is based on image segmentation, which plays an important role in image analysis and the computer vision system. The image segmentation is separating objects, in this case printing elements, from the background. Among all segmentation techniques, the automatic thresholding methods are widely used because of their advantages of simple implementation and time savings. The basic idea of automatic thresholding is to automatically select an optimal, or several optimal, gray-level threshold value/s for separating objects of interest in an image from the background based on their gray-level distributions. For image segmentation, the simple and effective Otsu method was used. The Otsu method is one of the thresholding methods that calculates the optimum threshold by separating objects and background so that their combined spread is minimal.

The Otsu method computes a global threshold (level) that can be used to convert a real-time image to a binary image. The level is a normalized intensity value that lies in the range [0,1]. The Otsu method chooses the threshold to minimize the intraclass variance of the black and white pixels by applying an automatic threshold in order to efficiently segment the image; it is based by a discriminant criterion to optimize the function of separation of obtained classes in gray levels. This method is described very briefly:

Supposing every pixel forms an image represented by gray levels [1,2,‥,L], the number of pixels at level L is denoted by n_i_ and the total number of pixels by N. The gray-level histogram is normalized regarding a probability distribution expression:

(1) $$p_{i}=n_{i}/N,\;\overset{L}{\underset{i}{\text{∑}}}p_{i}=1$$

Assuming a classification of pixels in two classes C_0_ and C_1_ (objects and background) by a threshold level k, where pixels with levels [1,…k] belong to C_0_ and pixels with levels [k + 1,‥,L] belong to C_1_. The probabilities of the class occurrence and class means levels are written by:

(2) $$\begin{array}{c} w_{0}=\mathrm{Pr}(C_{0})=\overset{k}{\underset{i=0}{\text{∑}}}p_{i}\\ w_{1}=\mathrm{Pr}(C_{1})=\overset{L}{\underset{i=k+1}{\text{∑}}}p_{i} \end{array}$$

(3) $$\begin{array}{c} \mu_{0}=\overset{k}{\underset{i=1}{\text{∑}}}i\mathrm{Pr}(i|C_{0})=\overset{k}{\underset{i=1}{\text{∑}}}ip_{i}/w_{0}\\ \mu_{1}=\overset{L}{\underset{i=k+1}{\text{∑}}}i\mathrm{Pr}(i|C_{1})=\overset{L}{\underset{i=k+1}{\text{∑}}}ip_{i}/w_{1} \end{array}$$

In this step, we are ready to define the following relation for the choice of k and the variance based on first order statistics (class means):

(4) $$\mu_{T}=w_{o}\mu_{0}+w_{1}\mu_{1};w_{1}+w_{o}=1$$

(5) $$\sigma_{B}^{2}(k)=\frac{{\left[\mu_{T}w(k)-\mu (k)\right]}^{2}}{w(k)[1-w(k)]}$$

The optimal threshold k* that maximizes σ_B_^2^ is selected by this sequential search using the cumulative quantities expressed in Equations (1) and (2):

(6) $$\sigma_{B}^{2}(k*)=\underset{1\leq k\leq L}{\text{max}}\sigma_{B}^{2}(k);\;\text{S}*=\left\{k;\;w_{0}\;w_{1}=w(k)[1-w(k)]>0\right\}$$

The result of the image segmentation is an image in binary mode, converted to black and white pixels, which differentiates the printing element from the background. The second step includes cropping and binarizing images by applying circular and rectangular windows at the central point of the input image. The circular and rectangular windows were applied 10 times; each time, the size of the window was changed to keep proportion with the size of the image. In the last step, the algorithm counted the black and white pixels on the 10 circular and rectangular images while determining the main value of the 10 results.

#### 3.1.2. Second Strategy (Printing Element Extraction and Quantification)

The *printing element extraction and quantification method* is more complex than the *binarized quantification method*. It consists of six different steps to obtain results for the printing plate quality. The image is captured by means of a microscope-based device; because of the deficiencies in this way of capturing, noise appears on the edges of the images. To avoid unwanted noise, the real-time image is cropped in a rectangular shape, keeping proportion for width and height with the real image. The next step is binarizing the image, which means to create image segmentation using the Otsu method, previously explained. The Otsu method computes a global threshold (level) that can be used to convert a real-time image to a binary image, normalizing the intensity value in the range [0,1]. The Otsu method chooses the threshold that minimizes the intra class variance of the black and white pixels.

The third step is edge detection using a Sobel operator, which is used in image processing, particularly in edge detection algorithms. The Sobel operator is an algorithm which clearly discovers the boundaries between regions in an image. It works on the basis of the image gradient, which is a change in intensity (or color) of an image. An edge in an image occurs when the gradient is greater, and the Sobel operator makes use of this fact to find the edge in an image. The Sobel operator calculates the approximate image gradient of each pixel by convolving the image with a pair of 3 × 3 filters. The filters estimate the gradients in both horizontal and vertical directions.

The next step utilizes the Hough transform, which is a method for detecting curves by exploiting the duality between points on a curve and the parameters of that curve. The process of identifying possible circular objects is relatively simple. The Hough transform technique first creates accumulator space, which is made of a cell for each pixel. For each edge point in an image, each pixel calculates the right radius. The calculating stops when the algorithm finds a center for the circle that is the intersection of all circles that centers on the edge of an object.

In the fifth step, the algorithm defines the threshold, the boundary which separates correct circles from incorrect. The threshold is defined by an equation which is related to an actual circle (candidate) and the circle which is following the actual:

(7) $$\text{Threshold}=\frac{r^{2}}{{\left(r+1\right)}^{2}}$$

If the threshold is lower than the coverage ratio, the circle is not acceptable, and the system removes that circle. This step is necessary to eventually remove noise on the binary images to obtain quality results.

After this final step is complete, all circles which represent printing elements should be recognized by the algorithm. If any circle is not recognized, the algorithm calculates the distance between the centers of two neighboring circles. Besides calculating distance, the algorithm also calculates the angle so that the system can replace any missing circle.

After all steps have been completed, the final quality measure is corrected by means of calibration approach. It is performed a lineal correction from the measure of the polynomial curve Energy based Hough and it is done a linear based correction from this measure (Pα + β). Being P the polynomial fitting obtained taking into account the energy of Hough transform.

**Figure 7.**
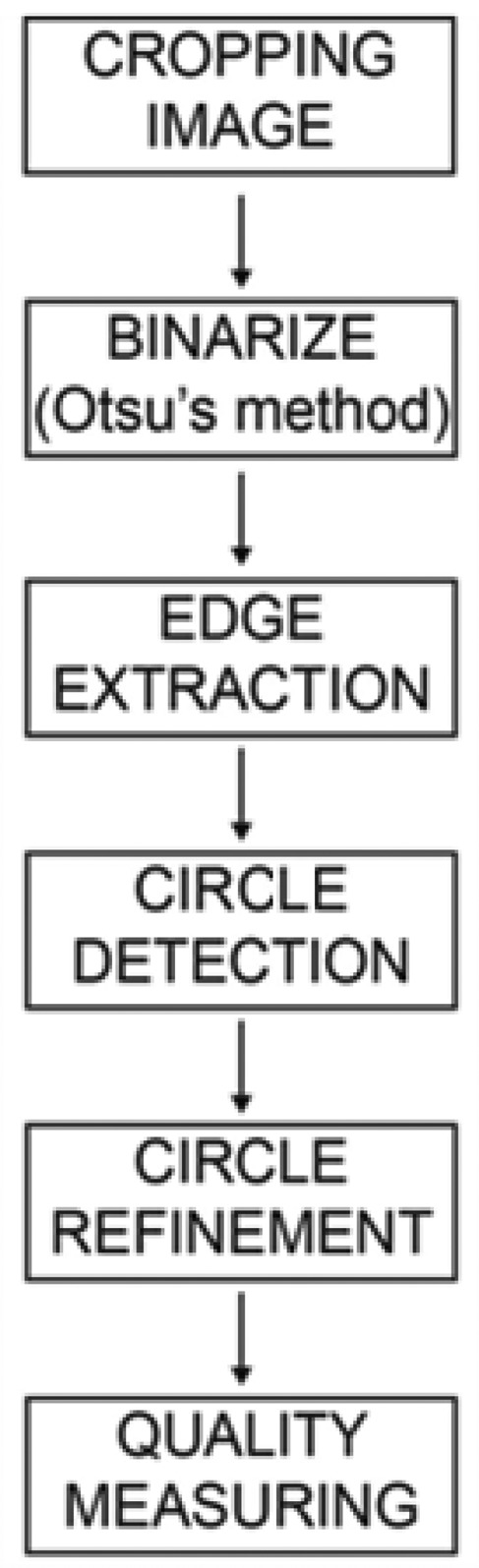
Flow chart from the second method implemented.

A summary of the flow chart can be found in Figure 7.

In the whole process, the Hough transform is the most important stage, since it is responsible for obtaining the circle centers and their radii. The Hough transform is based on the assumption that circles have a particular R radius. If the equations of each circle are:

(8) x=a+Rcosθ

(9) y=b+Rsinθ

Every point in the (x,y) space will be equivalent to a circle in the (a,b) space (R is not just a parameter, since it is known). Thus, when the equations are rearranged:

(10) $$a=x_{1}-R\cos\theta$$

(11) $$b={\text{y}}_{1}-R\sin\theta$$

for a particular point (x_1_, y_1_), and θ sweeps from 0 to 360 degrees. The brighter the spot, the more votes at the point. More votes imply a greater probability of a point being a center.

**Figure 8.**
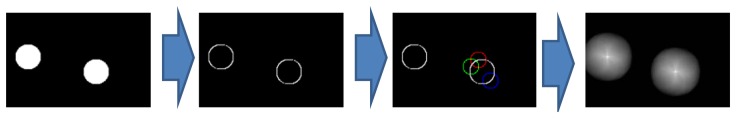
Scheme of the Hough transform.

Figure 8 describes the Hough transform process graphically. Each edge point is at the same distance from the center (R distance). If a circle of R radius is drawn for every point on the edge, a brightest point emerges corresponding to the center searched. In Figure 8, three random points were chosen and the corresponding circles were drawn (red, blue and green). Then, votes were cast at the pixels of these circles.

Using Hough transform terminology, the brightest spot corresponds to the highest energy pixel (most votes received). In the case of studying printing plates, a growing trend exists for higher energy pixels until the required R radius is found, which means the Hough method has already converged.

## 4. Results

### 4.1. Binarized Quantification

**Figure 9.**
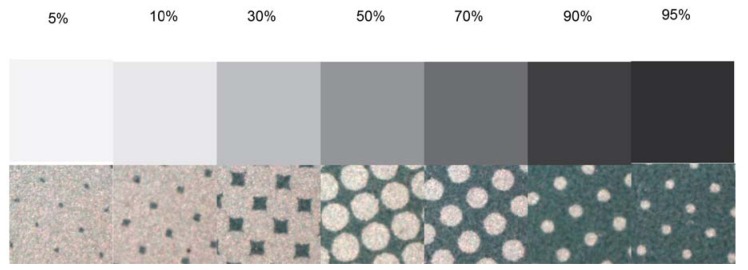
Control wedge *vs.* VipFLEX images.

**Figure 10.**
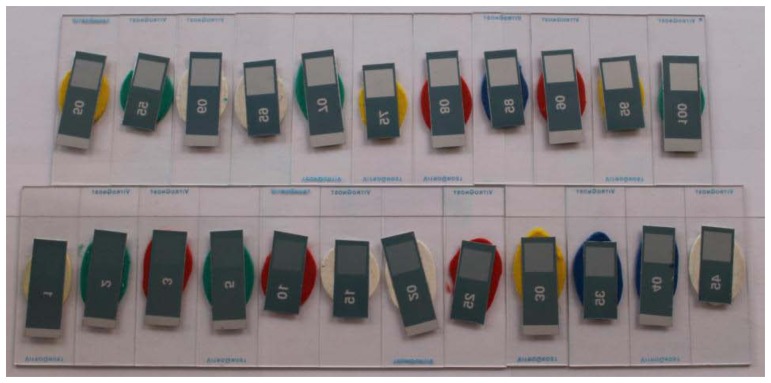
Printing plate samples.

Figures 9 and 10 correspond to the experimental printing plates used in this study. Meanwhile, Figure 9 shows the wedge view in comparison with the results obtained using the commercial system VipFLEX [24]. Figure 10 shows photographs with the 23 real printing-plates samples.

**Figure 11.**
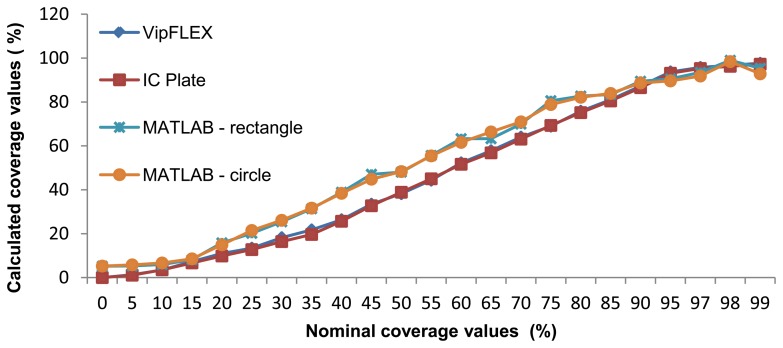
Comparison of nominal and measured coverage values obtained with different methods, 0% to 99%.

Results of the coverage value measurements were obtained by using six different measuring methods. For each sample, the mean value was calculated. The results can be seen in Figure 11. Nominal values correspond to each printing plate label. Columns reflected as VipFLEX and IC Plate II correspond to the existing commercial systems. Matlab was used to measure both software systems using different cropping values and methods (circular and rectangular). The mean values of surface coverage represent the reproduction of an entire tone scale. Results calculated using Matlab differ from other methods used to determine coverage calculation. The deviations can be explained in three areas.

The mean values of surface coverage are shown in Figure 11, which represents the reproduction of an entire tone scale. Results calculated using Matlab differ from other methods used to determine coverage calculation. The deviations can be explained in three areas.

The first deviation is visible up to 15% coverage (highlights), where Matlab results are higher than others. The second deviation is visible from 20% to 90% coverage, where Matlab results are higher than others, too. And finally, the third deviation can be seen in shadows (over 90%). There are two points of quality value calculated from all methods: at about 15% and 90% coverage.

Figure 11 shows the results of surface coverage values from the first half of the tone scale obtained with six methods. One can see that there are deviations between methods, especially between results measured with devices and results calculated with the image analysis software. Results obtained with VipFLEX and IC Plate II have the same results from 0% to 15% coverage. There are deviations in the results between 15% and 40% coverage, which means that these areas cannot be measured accurately. After 40% coverage, the results measured with the devices are the same. In the first half of the tone scale, deviation exists between the image analysis software and the devices, with one exception: the results are the same at 15% coverage. The possible reason for those deviations is in the usage of real images for printing plate samples. The images captured had round noises on the edges because of the way they were captured. The deviations between image analysis software and measuring devices can be seen up to 90%, after which results are quite similar.

As showed in the graphic diagrams, there are deviations between nominal and measured coverage values obtained with different methods. The results most similar to the nominal values are results obtained with the IC Plate II and VipFLEX measuring devices. Coverage values obtained with image analysis software (Matlab) show, to a certain extent, the deviations because the quality of their results depends on the quality of the sample images used for calculation.

Table 1 presents the calculation of the correlation coefficient, based on the Pearson model. According to the results, the correlation is very high (approximately 1) for all observed methods for calculating the surface coverage. This means that the application of any of these methods can provide reliable results. However, in the graphic diagrams, deviation is obvious in measured coverage values, especially between 15% and 90% (Figure 11).

**Figure 12.**
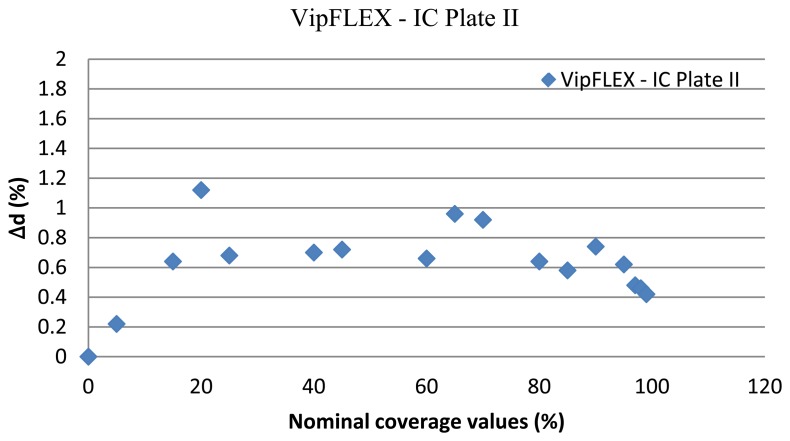
Comparison of coverage values calculated by VipFLEX and IC Plate II.

For further analysis of results, the standard deviation was calculated. Since VipFLEX and IC Plate II devices are designed for measuring the quality of printing plates, the standard deviation between their results were calculated first. The results are shown in Figure 12, where *Δd* represents the difference between coverage values measured by the IC Plate II and VipFLEX:

(12) Δd=coverage value(IC Plate II)−coverage value(VipFLEX)

Note that the correlation factor is less than 1% for all cases, meaning that the present prototype reaches the same results as commercial devices, so for further calculations, the method used is not particularly important. Since the IC Plate II device is specialized for offset printing measurements, it will be used in further calculations here, as the baseline. There is no appreciable difference between either cropping method (the correlation factor difference is negligible), which suggests the quality metric result is independent of the window chosen. Because of this, rectangular cropping was used since it is faster and more computationally affordable.

As we can see in Figure 12, the correlation coefficient is high (0,9998). From these results, it can be concluded that there are no differences in the calculation of surface coverage obtained by those devices. They can be used separately in the analysis of offset printing plate quality and will yield the optimal and same result of coverage. Since the IC Plate II device is specialized for offset printing measurements, these results were used in further calculations.

**Figure 13.**
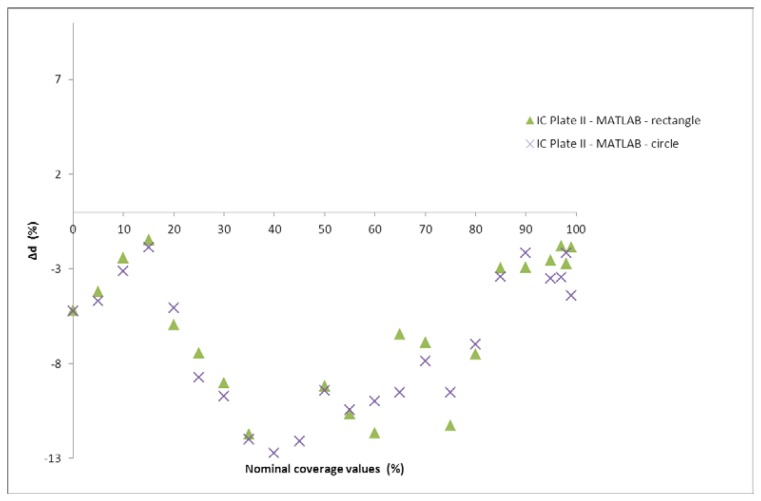
Comparison of coverage values calculated by IC Plate II and Matlab (circle and rectangle).

Figure 13 shows the difference between coverage values measured by the IC Plate II and calculated with Matlab (circle and rectangle). It can be seen that certain differences are present, as was expected since the results of the coverage presented in Figure 11 showed similar differences. Matlab was used for calculation of surface coverage based on image analysis.

### 4.2. Printing Element Extraction and Quantification

The main objective of this second strategy is to create an expert system which can determine the original circles plate and thus assess the strain used. It is an improved approximation of the first strategy that incorporates quality measures such as the resolution, the distance between printing elements, the screening angles, and the element deformation. The following items summarize the steps involved in this strategy, which were described in detail in Section 3.1.2.

**Figure 14.**
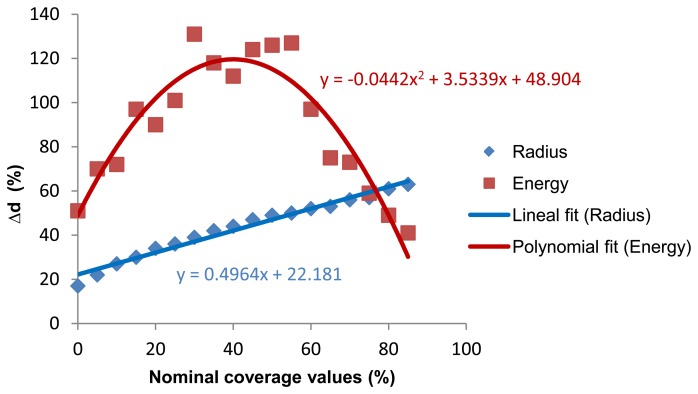
Radius and energy obtained for the different printing plates.

(1) Initially, the image is cropped in order to eliminate possible aberrations due to the lens in capture processing.
(2) The cropped input image is then segmented by means of the Otsu method.
(3) Circle edges are extracted using the Sobel operator.
(4) The Hough transform is performed, which allows the circle centers and their radiuses to be caught.
(5) An extrapolation mythology of resulting centers is completed, and the final quality analysis of the resulting image is performed taking into account a calibration approach: It is fitted the energy by polynomial curve (see Figure 14) and it is performed a linear correction from this measure Pα + β. Where P is the polynomial fitting obtained taking into account the energy of Hough transform and α and β are empirical values as follow α = 5/11 and β = 30.

The Hough transform is the core of this approximation, which allows the unknown radius to be extracted by energy measurement (Hough's accumulator). The higher the energy index determined, the more accurate the radius acquired, the higher the probability of a center being discovered.

Figure 14 shows the correlation between the radius and energy. It is evident that the radius will grow as the coverage value increases. Energy is higher for middle coverage values, while in the highlights and the shadows it is lower. The reason for the polygonal trendline comes from the energy results; the fact is that the system detects the background as a printing element instead of circles after 50% coverage.

**Figure 15.**
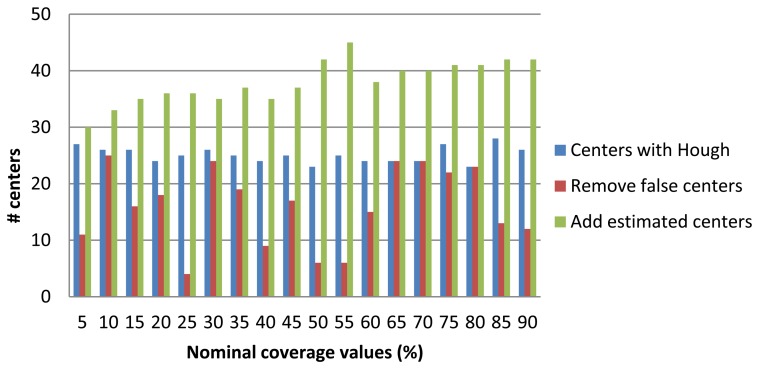
Number of centers obtained with Hough (blue bar), after false centers removal (red bar) and final estimation of new centers (green bar).

**Figure 16.**
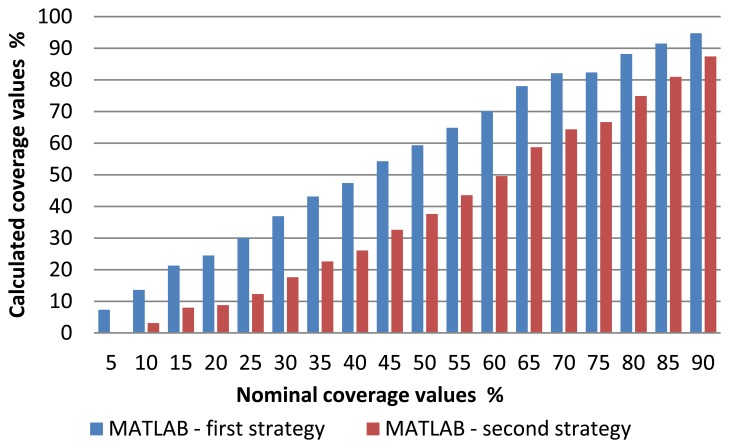
Results of total coverage obtained with the first and second strategy.

After obtaining the candidate centers, it is proceeded to eliminate those we consider invalid respect to a quality metric. This metric corresponds to a threshold value equation described (7). Figure 15 shows on one hand the number of centers obtained with the Hough transform (in blue bars) and those that remain after applying the aforementioned quality metric (in brown bars). Finally, we estimate the centers missing to complete the picture by generating a regular pattern extrapolating the distance between centers and their orientations. The total number of centers, either estimated or those remained from the previous phase are shown with the green bar. Finally, Figure 16 highlights the results of the total coverage for the first and second strategy used.

**Figure 17.**
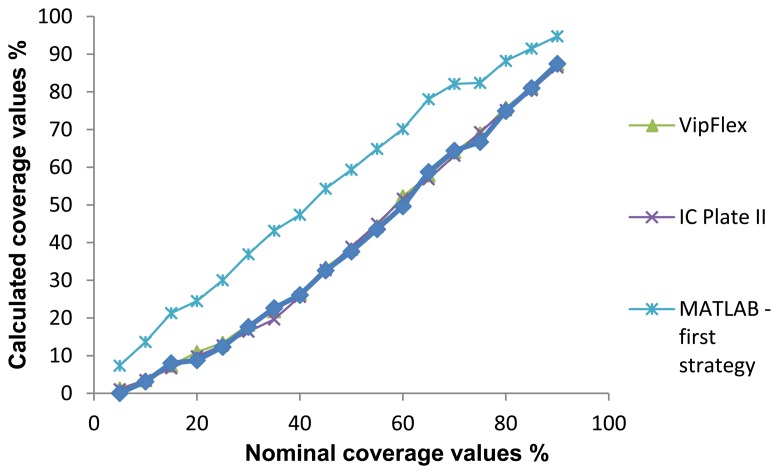
Comparison of nominal and measured coverage values obtained with different methods.

Figure 17 presents a comparison between different methods including the “Matlab-second strategy” which yields the surface coverage values. When viewing the comparison graphic diagrams, the deviation between the Matlab-second strategy and other methods used becomes evident.

### 4.3. Implementation in a Low-Cost Device

In the previous section, the advantages of our proposed system are shown. A feasibility study for its implementation in the low-cost devices outlined as follows. The motivation behind this study is supported by the possibility of improving industrial printing systems with an embedded device that regularly tests for degradation of quality printing plates. This improvement enables the continuous operation of printing plates by removing the need for technical stops in order to evaluate the quality. Under this premise, the methodologies described in previous sections are implemented in a low-cost device.

A low-cost candidate is a Raspberry-Pi board [26], based on an embedded ARM processor that can be purchased for less than $25.The ARM architecture is based on a 32-bit RISC processor, designed and licensed by the British company ARM Holdings (Cambridge, UK). The first design dates back to the 1980s, however the chip used in the vast majority of mobile devices today represents 95% of smartphones, 35% of digital televisions and set-top boxes, and 10% of mobile computers. The relevance of this processor is well demonstrated by net sales in 2010 with more than 6.1 billion shipments of ARM-based processors.

**Figure 18.**
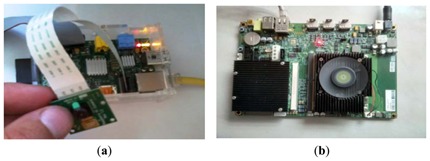
Scheme of the low-cost system. (**a**) Raspberry Pi and (**b**) A Carma-board.

Raspberry Pi has a Broadcom BCM2835 System on a chip, which includes an ARM1176JZF-S 700 MHz processor and a VideoCore IV GPU with up to 512 MB of main memory. Raspberry Pi's distributors also offer an inexpensive camera board with a resolution of 5 megapixels, which allows HD video recording. In Figure 18a, a scheme of a low-cost prototyping board is shown that contains the ARM1176JZF-S processor to be programmed. In order to evaluate a more powerful system, we have also considered a Carma-DEVKIT from SECO company [27] that integrates a CPU and Nvidia-Tegra3 with a Quad-Core ARM Cortex A9. In Figure 18b the Carma board that contains the ARM Cortex A9 processor is shown.

Table 2 summarizes the main features of the systems used in this paper. As a comparison, we also include the processor ARM-Cortex A57, considered the most powerful ARM processor, expected to be available by mid-2014. Table 3 compares peak performance, the number of available cores, and the overall cost of the system, except in the powerful ARM Cortex A57, whose prototype is not yet on the market.

As an addition to the study presented in previous sections, further discussion of the implementation of the sensor is warranted, focusing on the Raspberry Pi system. Image capture is carried out by means of the on-board camera available for this low-cost system (more information is available at http://www.raspberrypi.org/camera). Specifically, an implementation of the algorithm described in Section 3.1.2 has been ported into C/C++ high-level language that can be compiled with the native compiler GNU-GCC v4.6.

Our implementation efficiently exploits the ARMv6 architecture of ARM1176JZF-S processor. Most of the operations carried out are performed in integer arithmetic in order to avoid costly floating-point operations which offer poor performance in this architecture, except in Hough transform which is more sensible to accumulative round error.

**Figure 19.**
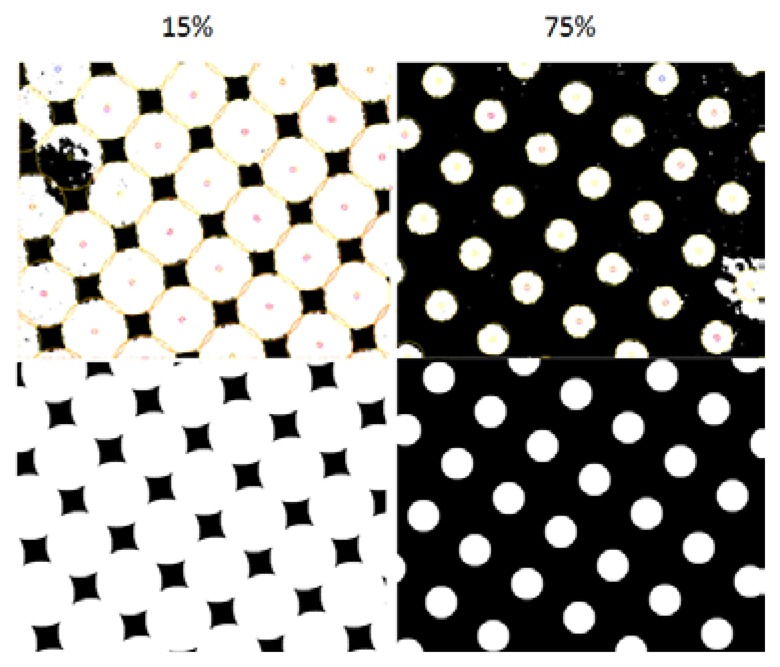
Noise part before and after sensor performing.

Figure 19 shows the visual outputs provided by our developed prototype. As is evident, the integer arithmetic used brings an accuracy solution without appreciable errors. Also remarkable is that the robustness of the system, illustrated in the lower part of Figure 19, shows that this approach is capable of eliminating dust in the acquisition of information and extrapolating circles in those areas which are not a priori. Also remarkable is that for the sample considered, plates with 15% and 75% surface coverage, the robustness system achieves dust removal in the acquisition phase, seen in Figure 19. The system is also able to interpolate new elements on noise areas.

**Figure 20.**
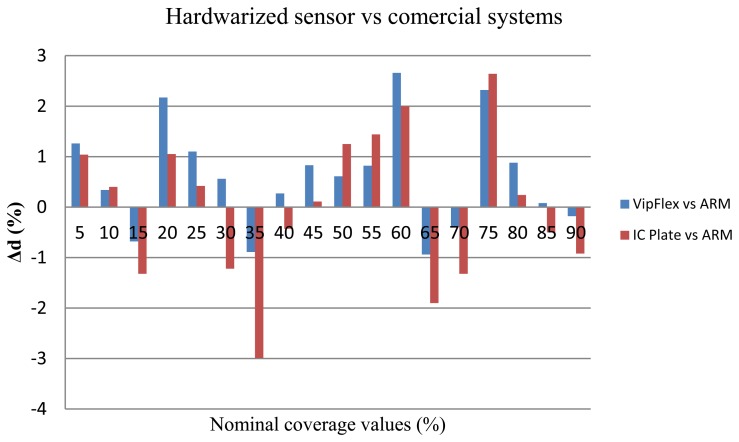
Comparison of nominal and measured coverage values in the hardwarized sensor based on ARM.

Regarding the accuracy of the system to provide quality metrics, Table 3 also presents the correlation coefficient based on the Pearson model in comparison with commercial systems. According to the results, the correlation is very high (approximately 1) for all observed methods for calculating the surface coverage. This fact means that application of any of these methods can obtain reliable results. Moreover, Figure 20 presents the difference between coverage values measured by the IC Plate II, VipFLEX, software implementation with Matlab, and our hardwarized sensor implementation based on the ARM processor. Focusing just on this image, the deviation between commercial devices and the ARM implementation is a curve with variable values as a peak of 3%, with an error mean of 1.2%:

(13) $$\Delta d_{VA}=\text{coverage value}\;(\text{VipFLEX})-\text{coverage value}\;(\text{ARM})$$

(14) $$\Delta d_{PA}=\text{coverage value}\;(\text{IC Plate II})-\text{coverage value}\;(\text{ARM})$$

#### 4.3.1. Time Performance in a Low-Cost Device

**Figure 21.**
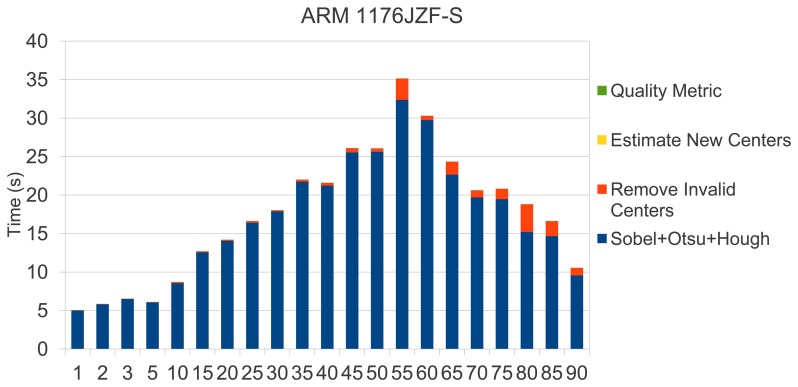
Execution time in Raspberry Pi's system (ARM 1176JZF-S processor).

In this section, we present a time performance study of our sensor. Figure 21 shows the sensor processing times observed in the system mentioned above. As shown, in all cases a quality test can be performed in less than 35 s, which supposes a reasonable time in a printing scenario if the printing system requires stopping to update printing plates. Remarkable is that image acquisition involves less than 2 s at 2,592 × 1,944 pixels of resolution with an on-board Raspberry camera. Thus, our low-cost sensor is totally affordable in terms of response time.

These remarkable response times of the sensor are mainly due to an efficient implementation based on the use of integer arithmetic that significantly speeds up arithmetic operations. The use of integer arithmetic at the expense of floating point arithmetic shows accuracy loss of 0.1%, which is more than a reliable rate.

Once the feasibility of new sensor implementation was addressed, a scalability study was accomplished. The main aim was to explore the chances of using a sensor based on a more powerful processor. The new proposed system (Carma board) consists of 4× ARM processors, so the task of parallelism could be exploited with an important reduction in execution time. A multithreaded application could be coded by means of OpenMP [28] standard programming.

OpenMP uses a portable, scalable model that gives programmers a simple and flexible interface for developing parallel applications for platforms ranging from the standard desktop computer to a supercomputer. OpenMP is an implementation of multithreading, a method of parallelizing, whereby a master thread (a series of instructions executed consecutively) forks a specified number of slave threads and a task is divided among them. The threads then run concurrently, with the runtime environment allocating threads to different processors. The programming is based on directives or tags, which eases the hard parallel coding task. The most common directive is #*pragma omp parallel for* which distributes the loop iterations between different threads. It should be noted that to achieve successful scalability rates, it is recommended to balance the workload among threads in a uniform way.

Before assessing the parallelization gains, it is worth mentioning that, as was expected taking into account the performance rates shown in Table 3, sensor response times were reduced by a factor of 2× with the use of a more powerful system (one ARM Cortex A9 processor). This means that in the worst-case scenario, the hardwarized sensor based on a single ARM-Cortex A9 would respond in less than 20 s.

**Figure 22.**
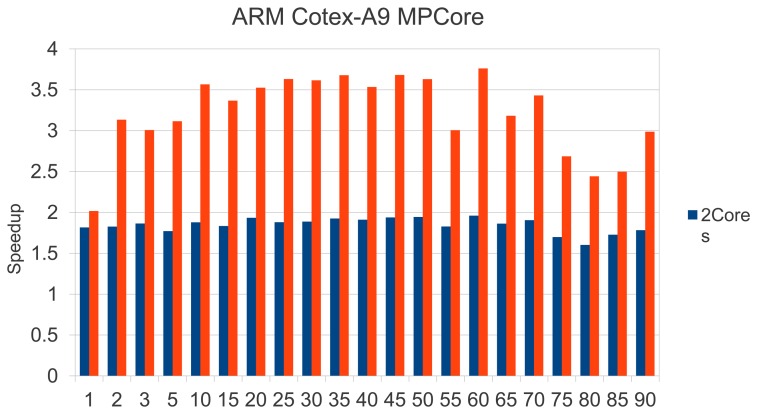
Speedup attained for 2 and 4 ARM Corex A9 processors.

Since the Hough transform is the most costly phase (see Figure 21), we focused on parallelizing the most demanding loops in Hough processing. Although the way to exploit parallelism is quite obvious, scalability is successful. We found an overall efficiency rate of 92% for two cores and 81% with 4 Cores. Figure 22 reinforces this fact, emphasizing the independence of the printing plate chosen. We would like to remark that the Carma board offers an enhanced performance rate, which supposes quality estimation times ranging between 2–5 s.

**Figure 23.**
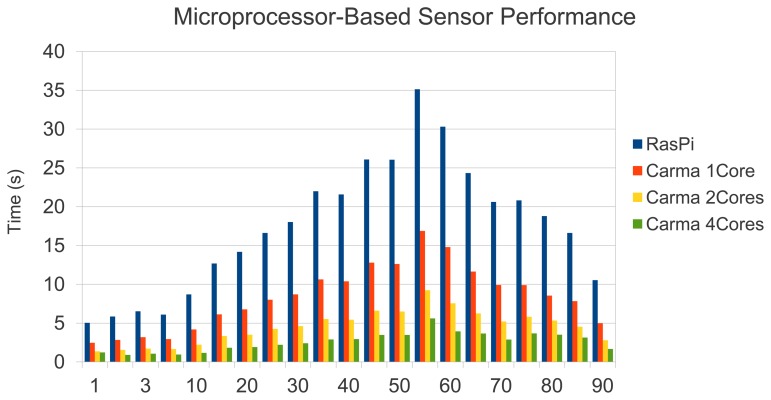
Summary of sensor times using different approaches (Raspberry-Pi and Carma board).

Finally, Figure 23 summarizes the performance behaviour observed, which shows the response times for the system based on the Raspberry Pi (blue bars), and Carma's kit performing 1 core (orange bar), 2 cores (yellow bar),and 4 cores (green bar).

### 4.4. Visual Results

**Figure 24.**
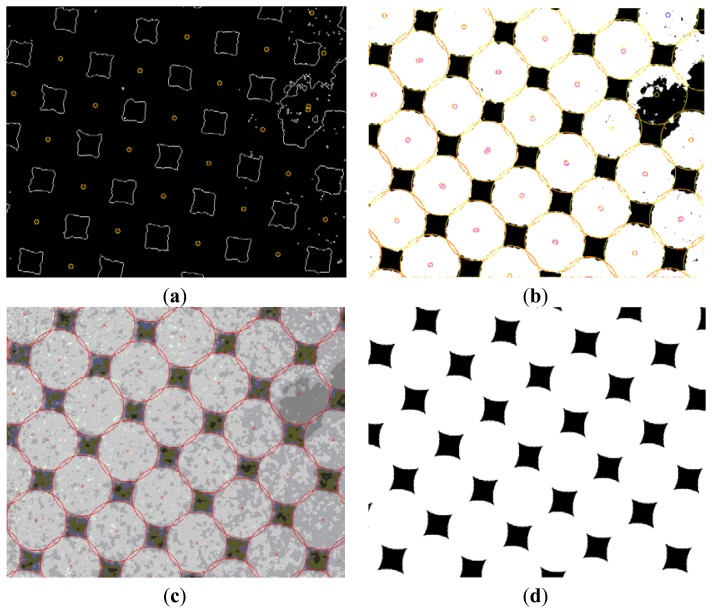
(**a**) Examples of edge circles detected for the first stages of our hardwarized sensor; (**b**) Examples of circles detected for the next stages of our hardwarized sensor. Note how the system is able to detect circle degradation and restore/measure the damaged surface; (**c**) Examples of circles overlapped with the real image; (**d**) Examples of circles, the real image recovered (nominal area 75%).

Finally, this subsection shows a visual sequence (Figure 24a–d), which displays the intermediate results for a particular printing plate sample with a nominal coverage area of 75% after performing:

(1) The transform of the detection axes by Sobel's method (Figure 24a)
(2) The centers detection by the Hough transform (Figure 24b)
(3) The interpolation of the circles not previously detected (Figure 24c)
(4) The quality estimation using the solution obtained (Figure 24d). Note that the image captured has some noise, which makes circle detection difficult. However, as shown in the sequence of images, our low-cost sensor is sufficiently robust to allow for a certain degree of ambient noise.

## 5. Conclusions

There are currently few quality control methods for printing plates, but the image analysis method proves to be the best. This method uses microscopic images of the printing plate surface and software to analyze these images. The image analysis software shows a percentage of the surface coverage with the printing elements.

This work describes the implementation of a hardwarized sensor based on a low-cost system especially designed to measure the quality of printing plates. The main problem was previously evaluated using two strategies: (1) those based on dynamic histogram segmentation; and (2) those based on the previous steps plus the Hough transform and refinement in order to construct a robust machine vision system able to deal with noise and other drawbacks in the real environment. These algorithms have been implemented using a specific approach based on low-cost processors similar to mobile devices, such as tablets, smartphones, and so on. Meanwhile the existing commercial devices cost around 3,000–5,000 US dollars; our prototype does not reach $150, which, due to its low cost, opens up the chances for incorporation inside printing systems as an alert when certain printing plates wear out.

Our results have shown that our hardwarized sensors are also able to detect and visualize motion artifacts with a high rate of accuracy. Currently, our system provides a robust and complex Printing Plate quality measurement in a time-set between 5 s to 30 s, competitive against fully scalable and modular commercial devices with an error *versus* commercial devices less than 3%. We are currently further improving the system with the hierarchical multi-scale optical flow algorithm, and we will evaluate the achieved motion correction based on a Receiver Operating Characteristic (ROC) over different embedded GPUs in order to export that to mobile devices as well.

In this paper, the surface coverage was measured as a main quality metric using different methods, but our sensor approach also considered other metrics, such as circle deformation, the distance between printing elements, or the screening angle. Captured images from the printing plate surface using circle printing elements were inserted into the software. Future experiments will consider how to measure surface coverage when the elements are not circle-shaped (for example, oval) or when printing elements have special design shapes.

In offset printing, every color uses a printing plate for itself with printing elements placed at different angles to avoid the appearance of a moaré pattern. Accordingly, for future work, the software made in Matlab could make sure if the printing elements for different colors are under the corresponding angles.

Furthermore, developing this software and making it suitable for other printing techniques is also one of the possibilities for future work. Additionally, linking software with appropriate hardware could result in prototypes which could define the quality of printing plates in a new way.

## Figures and Tables

**Table 1.** Pearson correlation coefficient.

**Correlation**	**Correlation Coefficient**
VipFlex *vs.* IC Plate	0,9998
IC Plate *vs.* MATLAB (rectangle)	0,9909
IC Plate *vs.* MATLAB (circle)	0,9906
VipFlex *vs.* MATLAB(rectangle)	0,9908
VipFlex *vs.* MATLAB(circle)	0,9907
MATLAB (circle) *vs.* MATLAB (rectangle)	0,9994

**Table 2.** Summary of features from systems used.

**Board Name**	**Processor Used**	**Performance(DMIPS^1^)**	**# Cores**	**Cost**
Raspberry-Pi	ARM1176JZF-S	965	1	<40€
Carma DEVKIT	ARM Cortex A9 MPCore	5,000 per core	4	529€
	ARM Cortex A57 MPCore	20,000 per core	1–16	

1 Dhrystone MIPS: synthetic benchmark developed in 1984 that represent integer performance power of a computer in Millions of Instruction Per Second (MIPS).

**Table 3.** Pearson correlation model for the second strategy.

**Correlation**	**Correlation Coefficient**
VipFLEX *vs.* ARM	0.9993
IC Plate II *vs.* ARM	0.9987
